# Robot Faces that Follow Gaze Facilitate Attentional Engagement and Increase Their Likeability

**DOI:** 10.3389/fpsyg.2018.00070

**Published:** 2018-02-05

**Authors:** Cesco Willemse, Serena Marchesi, Agnieszka Wykowska

**Affiliations:** Social Cognition in Human-Robot Interaction, Istituto Italiano di Tecnologia, Genoa, Italy

**Keywords:** gaze-leading, gaze-following, gaze-cueing, joint attention, human–robot interaction

## Abstract

Gaze behavior of humanoid robots is an efficient mechanism for cueing our spatial orienting, but less is known about the cognitive–affective consequences of robots responding to human directional cues. Here, we examined how the extent to which a humanoid robot (iCub) avatar directed its gaze to the same objects as our participants affected engagement with the robot, subsequent gaze-cueing, and subjective ratings of the robot’s characteristic traits. In a gaze-contingent eyetracking task, participants were asked to indicate a preference for one of two objects with their gaze while an iCub avatar was presented between the object photographs. In one condition, the iCub then shifted its gaze toward the object chosen by a participant in 80% of the trials (joint condition) and in the other condition it looked at the opposite object 80% of the time (disjoint condition). Based on the literature in human–human social cognition, we took the speed with which the participants looked back at the robot as a measure of facilitated reorienting and robot-preference, and found these return saccade onset times to be quicker in the joint condition than in the disjoint condition. As indicated by results from a subsequent gaze-cueing tasks, the gaze-following behavior of the robot had little effect on how our participants responded to gaze cues. Nevertheless, subjective reports suggested that our participants preferred the iCub following participants’ gaze to the one with a disjoint attention behavior, rated it as more human-like and as more likeable. Taken together, our findings show a preference for robots who follow our gaze. Importantly, such subtle differences in gaze behavior are sufficient to influence our perception of humanoid agents, which clearly provides hints about the design of behavioral characteristics of humanoid robots in more naturalistic settings.

## Introduction

Robotic agents are increasingly advanced from a technological perspective, which provides an excellent opportunity to apply methods and techniques from social cognition research to examine the aspects of social interaction between humans and robots (Wykowska et al., 2016; Wiese et al., 2017) – an important research question to be asked when facing the new era of social robots soon inhabiting human environments. Robots are an interesting case of agents that might fall in the category of a tool or of an animate object (e.g., Brown and Goodale, 2013). Measuring the degree to which mechanisms of social cognition are activated when interacting with humanoid robots brings us closer to understanding the conditions under which robots are perceived as animate agents. Here, we examine whether patterns of gaze-directed dyadic human–robot interactions match those we typically see in social attention toward human faces, whether they have consequences for subsequent engagement in joint attention, and whether they affect how these robots are perceived.

In human–human interaction, one fundamental mechanism of social interaction is joint attention, which is established when one person orients attention toward an object, the other person follows, and they are now attending to the same object due to the first person’s original attentional signal (Emery, 2000). A strong attentional orienting signal in establishing joint attention is gaze direction (Langton et al., 2000). In the literature, paradigms with manipulations of gaze directions of a face on the screen have been described to underpin the mechanisms of following – or *responding to* – gaze, as well as *initiating* joint attention with gaze (Frischen et al., 2007; Mundy and Newell, 2007; Bayliss et al., 2013). One prominent method to investigate responding to gaze is the gaze-cueing task (Friesen and Kingstone, 1998; Driver et al., 1999), a modified version of the Posner (1980) cueing paradigm.

### Responding to Gaze

In the gaze-cueing paradigm, a face is presented in the center of the screen. At some point, the face diverts its eyes to the left or the right. After a certain time (stimulus onset asynchrony; SOA), a target is presented to the left or the right of the face. Thus, the cue provided by the face can be valid, when the target appears congruently with the face’s gaze direction, or invalid when the target appears contralaterally to the gaze cue. Participants, who are instructed to ignore the gaze direction and to identify the target, typically show quicker response times (RTs) to validly cued than to invalidly cued targets (for a review, see Frischen et al., 2007). This gaze-cueing effect is most prominent for SOAs between 100 and 300 milliseconds and typically disappears after around 1 s (cf. Friesen and Kingstone, 1998; Friesen et al., 2004; Frischen and Tipper, 2004; Tipples, 2008). Further, gaze-cueing effects have also been found for schematic representations of faces (Friesen and Kingstone, 1998; Ristic et al., 2002; Frischen et al., 2007; Kuhn and Kingstone, 2009) and of particular interest also for robot avatars (Wiese et al., 2012; Wykowska et al., 2014) and embodied humanoid robots (Wykowska et al., 2015; Kompatsiari et al., 2017).

### Initiating Gaze

More recent research has examined the affective and cognitive effects of initiating joint attention through gaze-leading paradigms (Bayliss et al., 2013; Edwards et al., 2015). In gaze leading, gaze behavior of the avatar on the screen is contingent on the eye movements of the participant (Wilms et al., 2010). It has been found that initiating joint attention modulates implicit measures as well as explicit ratings of affect. For example, by combining eyetracking with fMRI methodologies, Schilbach et al. (2010) found that initiating joint attention activated the ventral striatum; an area related especially to the hedonistic and motivational aspects of reward experience (Liu et al., 2007; Rolls et al., 2008). Their participants also rated the experience of having their gaze followed by the avatar as more pleasant relative to not having their gaze followed. However, measures of pleasantness, or likeability, of the avatars themselves were not assessed. In a series of experiments, Bayliss et al. (2013) addressed this limitation among other implications of initiating joint attention by leading an avatar’s gaze. One of the aims of their study was to investigate gaze bias. Their experiments consisted of a face in the center of the screen and two laterally presented objects. Participants were instructed to form a preference for one of the two objects and press a key when a preference was reached. Next, upon hearing a cue, they had to look at the preferred object as quickly as they could to indicate their selection. Gaze dwell time during exploration of the objects while forming a preference predicted object selection, as reflected in the subsequent saccade to the preferred object as well as in later object ratings.

Importantly for the purposes of Bayliss et al.’s (2013) study, contingent gaze behavior of the avatars was also manipulated, with some identities always following the gaze of the participant and other identities always looking at the opposite object. First, gaze-following behavior of the avatar positively affected explicit pleasantness ratings of these avatars, thus complementing Bayliss et al.’s (2013) findings by showing that we do not only find the experience of initiating joint attention pleasant, but also find those agents with whom we establish it. Second, of particular interest are their findings in relation to the fact that participants were asked to return their gaze to the avatar to initiate the next trial. These return saccade onset times were slower when avatars did not follow the participant’s gaze than when they did. These findings appear to offer an implicit but reliable insight into how gaze behavior of those we interact with affects both how we perceive them and how we interact with them.

Other studies have found that this facilitated re-orienting of attention toward faces that followed participants’ gaze also occurs without explicit instructions to do so (Edwards et al., 2015) and that avatars who follow are also perceived as more trustworthy than those with whom initiated joint attention is not established (Dalmaso et al., 2016). It could be argued that these effects are a result of disjoint avatars looking in the opposite direction rather than being due to lack of joint attention. However, a study that controlled for this by implementing avatars with animated gaze behavior that either followed participants gaze or explored the same scene independently found similar effects of pleasantness, including when baseline ratings were considered, but less so for trustworthiness and closeness (Grynszpan et al., 2017).

From these studies, it remains unclear whether the delayed re-engagement with the face is driven by the learning of person information, i.e., “identity” (“this person never attends to the same object”) or of *ad hoc* mechanisms in response to online behavior (“this time a person attended to the other object”). In other words, are identities who never follow the gaze considered as norm violators in general, or does incongruent online behavior of the face indicate undermining the participant’s specific object choice? In order to examine this dissociation, we introduced an element of (un-)expectancy to the experiment presented herein. In a counterbalanced blocked design, we introduced our participants to two “different” iCub robots (Metta et al., 2010). In fact, we used the same image for these and provided no further information other than their names: Jimmy and Dylan. In the block where participants interacted with Jimmy, the robot displayed congruent following behavior in 80% of the trials and incongruent gaze behavior in the remaining 20% of the trials (joint attention disposition). For Dylan, this proportion was reversed (disjoint attention disposition). This allowed us to examine whether attribution of identity, or more specifically *disposition*, plays a role in the facilitation of return saccades alongside online behavior. Additionally, it allowed us to rule out that this would merely be an effect related to violation of expectations.

Speeded re-orienting after establishing joint attention relative to disjoint attention can be attributed to disposition and/or online behavior. Different underlying causes of this effect are possible. For example, faster reorienting could directly be explained in terms of pleasantness, cooperative status, or other such indirect attributions potentially associated with gaze following or establishing joint attention. In the literature highlighted above, establishing joint attention is positively correlated with post-experiment measures such pleasantness and trustworthiness, but the direction of this relationship remains relatively unclear. However, it is generally proposed that successful gaze-leading induces hedonistic reward mechanisms and likeability (Schilbach et al., 2010; Bayliss et al., 2013; Grynszpan et al., 2017). Alternatively, perhaps faster attentional allocation toward the avatar could be explained by its animacy: animate motion receives prioritized attention allocation compared with inanimate motion (Pratt et al., 2010). However, we argue that following and unfollowing gaze behavior of the avatar are equally animate. Finally, facilitated reorienting to the avatar could rather reflect an easier disengagement from the object if both avatar and participant “keep watch” of it (and therefore prolonged attentional allocation would be less crucial for the participant). In our study, finding a stronger effect of disposition than for online behavior would refute this possibility.

### The Impact of Initiating Joint Attention on Responding to Gaze

There is some evidence that initiating joint attention affects subsequent gaze-cueing with the same avatars (Dalmaso et al., 2016). In a broader sense, gaze-cueing effects have found to be modulated by the encoding of person information, such as facial features (e.g., Jones et al., 2010; Ohlsen et al., 2013) and facial expressions (e.g., Mathews et al., 2003; Kuhn and Tipples, 2011). Additionally, manipulations of social knowledge have also been shown to alter subsequent social interaction (Gobbini and Haxby, 2007; Todorov et al., 2007; Bayliss et al., 2012; Capozzi et al., 2016). Greater gaze-cueing effects have been observed for faces of known versus unknown individuals (Deaner et al., 2007), those with one’s matching political ideas (Liuzza et al., 2011), and of high compared with low social status (Dalmaso et al., 2011, 2014).

Whether gaze behavior of the responder to joint attention is sufficient to encode and retrieve such person information in later episodes of joint attention with these individuals is less clear. However, for example, it has been found that single exposures to a gaze cue by a specific identity affect the orientation of attention during a subsequent encounter with that identity (Frischen and Tipper, 2006). Of particular interest to the current study are findings from a series of experiments which examined how exposure to contingent gaze-responses of avatars influenced subsequent gaze-cueing (Dalmaso et al., 2016). The authors employed a saccade/anti-saccade task in response to peripherally presented targets while a face was presented in the center of the screen. Some of the faces also directed their gaze toward the target at which the participant was supposed to look, while other faces always looked away. Interestingly, subsequent gaze-cueing effects were less prominent for faces that followed the participant’s gaze, whereas faces who never followed elicited a consistent gaze-cueing effect. However, in the aforementioned study, participants were instructed to direct their gaze toward (or avert their gaze away from) a target with specific characteristics, without employing the free object preference and selection task in Bayliss et al. (2013). It is worthwhile to examine whether these influences on gaze-cueing are maintained when the avatar’s gaze direction could be interpreted as a violation of, or undermining, the participant’s choice. Therefore, we presented a gaze-cueing task after each block mentioned above.

### Aims of the Current Study

Our experiment consisted of two distinct parts: (i) a free object selection task in which the robot avatar’s gaze behavior was contingent on eye-movements made by the participant, and (ii) a task in which participants identified targets that appeared after the robot avatar redirected its gaze. In the literature, these tasks have been referred to as (i) “gaze-leading” and (ii) “gaze-cueing,” where the former is named from the perspective of what the participant does, but the second is named from the perspective of the avatar – who cues the participant’s locus of attention. To avoid potential confusion, we refer to these two parts of our experiment as “human initiator” and “human responder,” respectively, henceforth.

We aimed to investigate the following: we examined whether – in the human-initiator part – return saccades to a robot avatar have a faster onset when the avatar follows the participant’s gaze toward the same object, relative to when the avatar looks at the non-selected object. Because we manipulated gaze-following on two “levels”: per block and per trial, our study allowed to disentangle whether learned person information (“disposition,” per block) or online behavior (per trial) underpin the extent of facilitated return saccades. We hypothesized that participants would elicit facilitated return saccades toward robots that typically followed participants’ gaze, relative to those who did not. Additionally, we predicted that online behavior matters: regardless of the robot’s disposition, when the robot followed the gaze, participants would be quicker to return their gaze to it, compared with when it did not follow.

We also examined whether gaze-congruent behavior of the robot avatar affects subsequent responding to joint attention (human responder). After each human-initiator part, we presented a gaze-cueing paradigm with a reminder that participants would be interacting with the same robot as before. We cautiously hypothesized that participants would show a gaze-cueing effect for the robot in the disjoint condition but less so for the joint robot (as per Dalmaso et al., 2016). On the other hand, as gaze-cueing is found to be a robust effect (Frischen et al., 2007), we propose that perhaps this type of learned information is too subtle to greatly affect subsequent gaze-cueing. Finally, we explore whether robot’s behavior in the human-initiator part (following gaze or not) results in the typically following robot subjectively being perceived as more human-like and more likeable than a typically unfollowing robot, as well as explicitly more preferred by our participants.

## Materials and Methods

### Participants

A total of 32 participants (five males, mean age = 26.7; SD = 3.63) took part in the study in return for a payment of 15€. They were recruited through an internal mailing list. All participants self-reported normal or corrected to normal vision. Written informed consent was given by each participant. The study was approved by the local Ethical Committee (Comitato Etico Regione Liguria) and was conducted in accordance with the Code of Ethics of the World Medical Association (Declaration of Helsinki).

### Stimuli and Apparatus

The experimental session took place in a dimly lit room. Stimuli were presented on a 22′′ LCD screen (resolution: 1680 × 1050). A chinrest was mounted on the edge of the table, at a horizontal distance of 62 cm from the screen. Binocular gaze data was recorded with a screen-mounted SMI RED500 eyetracker with a sampling rate of 500 Hz. We used a 9-point calibration and validation (validation errors: *M* = 0.95°; SD = 0.54) prior to each task, and when deemed necessary (e.g., after two subsequent time-outs, at the experimenter’s discretion). The experiment was programed in and presented with OpenSesame 3.1.8 (Mathôt et al., 2012) using the PsychoPy 1.81 backend (Peirce, 2007, 2009) and the Pygaze library (Dalmaijer et al., 2014). For the presentations where gaze contingency was manipulated, we used a circular area of interest (AoI) with a radius of 1.3° in the center of the screen to determine the area in which participant’s gaze had to be in – to be considered valid and let the experiment move on.

A frontal image of the iCub’s face (7.67° × 7.67° was digitally edited to produce different gaze directions: straight, left, and right. These images were always presented in the center of the screen. For the human-initiator part of the task, photographs of 16 objects (variable size, mean size = 4.56° × 5.96°) were selected from a group of objects with which iCub can interact and then matched in eight pairs based on similar shapes and colors. For object with handles like a rake or a ladle, affordance was presented vertically. These stimuli can be found in Supplementary Figure 1.

In the human-responder part of the task the letters T and V were used as target stimuli (font: italic, size: 48). The objects in the human-initiator part and the target letters in the human-responder part, were presented 7.67° to the right and 7.67° to the left of the center of the screen. Order of block presentation and key mapping for the targets in the human-responder part were counterbalanced between participants. Responses were collected with a standard keyboard with two buttons identified as the ones to be used with stickers. Participants had to use both forefingers to answer (the left one on the left button, the right one on the right button). Stimuli presentation was randomized.

Three questionnaires were administered. First, the Godspeed questionnaire (Bartneck et al., 2009), then five additional questions regarding the experiment (Supplementary Table 1) and finally the Autism Quotient (AQ) questionnaire (Baron-Cohen et al., 2001). All questionnaires were presented in Italian (AQ and the Godspeed were translated from English).

The Godspeed questionnaire was created to investigate people’s perception of robots (Bartneck et al., 2009). Bartneck et al. (2009) identified five subscales (anthropomorphism, animacy, likeability, perceived intelligence, and perceived safety) rated on a 5-point scale. For this experiment’s interests, only the anthropomorphism and likeability subscales were used. At the end of each session, the experimenter asked the five additional questions and wrote down the participants’ answers. These questions were meant to investigate participants’ subjective perception of the robot’s behavior, focusing on preferences (if there was actually a preference) and differences (if there was an actual explicit perceived difference between the two robots). Finally, the AQ questionnaire (Baron-Cohen et al., 2001) was run on the screen to complete the experimental session. The decision to investigate the social aptitude (autistic-like) traits is justified by a series of studies which have shown interesting patterns between a high score on the AQ and the gaze behavior. Regarding responding to gaze, it has, for example, been found that a higher proportion of autistic-like traits is related to a smaller magnitude of the gaze-cueing effect (Bayliss and Tipper, 2005). In a gaze-cueing study with a robot face, autistic-like traits were inversely related to the participants’ sensitivity toward differences between human-like and pre-programed gaze behavior of the robot (Wykowska et al., 2015). Wiese et al. (2014) have shown a gaze-cueing effect for robot faces but not for human faces in individuals diagnosed with Autism Spectrum Disorder. In a gaze-cueing task presented after a gaze-following task, Dalmaso et al. (2016) found that those with more autistic-like traits were more cued by joint attention faces than those with lower self-reported autistic-like traits, but this correlation was not present in the condition in which the faces on the screen did not follow participants’ gaze. Regarding initiating gaze, it has been suggested with caution that perhaps having higher autistic-like traits is related to a weaker attentional orienting toward joint, or following, faces (Edwards et al., 2015). In order to further examine the potential modulation of autistic-like traits on initiating joint attention and on subsequent following as well as attribution of person information (disposition), we assessed these traits in our sample of participants.

### Procedure

Each participant was invited to sit in in front of the screen and to place their head comfortably on the chinrest. The experimenter introduced each participant to the eye-tracking procedure and instructed the experimental tasks: the human-initiator part of the task first (described to participants as “the object task”), and then the human-responder part of the task (described to participants as “the letter task”). These tasks were repeated for the joint/disjoint condition in a counterbalanced block design. Both tasks were instructed and practiced prior to the experimental session. All participants were instructed in Italian.

#### Human Initiator

**FIGURE 1:**
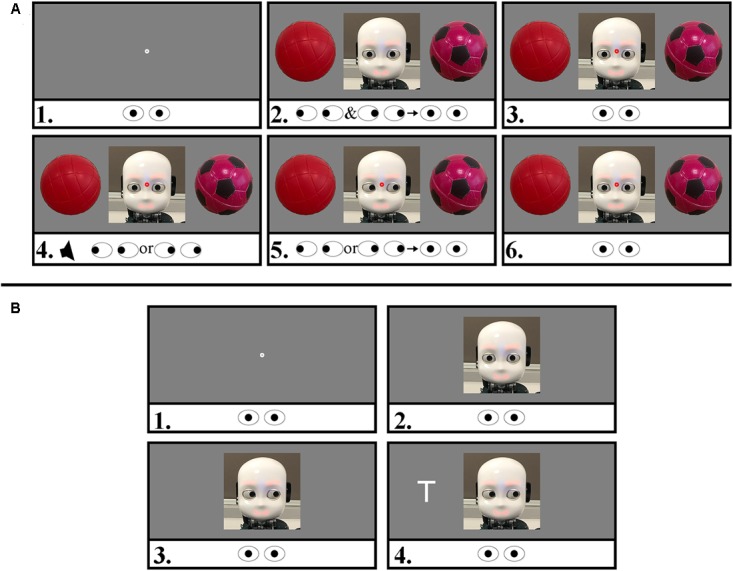
Trial sequences of both parts of the task with stimuli examples. The eye icons represent the required eye movements of the participant. Screen not drawn to scale. Panel **(A)**: human-initiator trial with one of the object pairs used in the experiment. 1. Gaze contingent fixation dot (500 ms). 2. iCub and objects appear. The participant is asked to explore the scene and fixate the robot face when a preference for one of the objects is decided. 3. A red fixation dot appears on the center of the face upon successful fixation. 4. 500 ms later, a beep sounds, prompting the participant to look quickly at the preferred object. 5. 300 ms after this saccade the robot follows or unfollows the participants gaze. Participants were instructed to look back at the robot in their own time. This return-saccade onset time is our DV of interest. 6. Upon fixation on the face, the robot avatar returns its gaze (500 ms, end of trial). Panel **(B)**: human responder. 1. Gaze contingent fixation dot (500 ms). 2. The iCub appears (fixate face, 500 ms). 3. Robot diverts gaze left or right (SOA 300 ms). 4. Target appears. Participants were asked to keep fixating on the face and identify the target, which was either a T or a V. Here, an invalidly cued trial is illustrated. The trial ended after a response was collected.

For the human-initiator part of the task, participants were told that they would interact with two different robots who would look the same in an object task, but had different personalities and names (Jimmy, for joint attention disposition, and Dylan, for disjoint attention disposition). Participants were not informed about the behavioral characteristic that differentiated the “personalities.” Trials in this part of the task started with participant looking straight to the center of the screen; after participant’s gaze was in the AoI, a contingent fixation dot in the center of the screen after which (500 ms) the iCub’s face would appear simultaneously with one object to its left and one to its right. Participants were asked to decide which object of the pair they liked best. They were asked to return their gaze to the center of the screen when they reached their decision. At this point, upon successful fixation on the robot’s face, a red fixation dot at the center of the iCub’s face was presented, followed after 500 ms by a sound cue (750 Hz, 100 ms). This cue indicated that participants had to move their gaze toward the chosen object as fast as possible. A total of 300 ms after this saccade onset, the robot directed its gaze to the same object, or to the one opposite, depending on the condition (Jimmy for “joint”: 80% times gaze-following; or Dylan for “disjoint”: 80% opposite). Participants were told to return their gaze to the center of the screen in their own time in order to initiate the next trial. The iCub returned its gaze toward the participant as soon as successful fixation hereon was established, see **Figure 1A** for an example trial.

Each human-initiator block consisted of 80 trials including a self-paced break after 40 trials. There were 20 practice trials for the human-initiator part of the task (10 with Jimmy and 10 with Dylan), which were similar to experimental trials with the following differences: In order to maximize the participant’s exposure to the typical behavior of each robot in this brief practice, Jimmy always followed the participants’ gaze, whereas Dylan always looked toward the opposite side, and the practice object pair was not used in the experimental trials.

#### Human Responder

For the subsequent human-responder part of the task, participants were told that they would continue to interact with the same robot as in the previous “object task.” Participants were instructed to the “letter task”: they were asked to stay fixated on the robot’s face (even if it would move its eyes) and identifying the letter (a V or a T) on one of the robot’s sides by pressing the corresponding button (left or right) on a standard keyboard, identified with stickers; keyboard mapping was counterbalanced between participants. Stimuli were presented in a randomized order between participants, with 50/50 validity. Participants were also told that robot’s gaze behavior was non-predictive about the letter position and that a tone would sound if they made a mistake (**Figure 1B**). The experimenter emphasized that the task should have been accomplished as fast and as accurate as possible. Each human-responder block consisted of 80 trials including a self-paced break after 40 trials. There were 8 practice trials, which were similar to experimental trials without the preceding instruction that participants would interact with a specific robot identity.

#### Questionnaires

The Godspeed (Bartneck et al., 2009) questionnaire was given after each block to rate the Anthropomorphism and the Likeability for each condition. The five additional questions were asked at the end of the experiment; the experimenter wrote down the participants’ answers. Finally, the AQ (Baron-Cohen et al., 2001) was run on the screen with EPrime (version 3). At the end of the session, participants were debriefed about the experiment.

### Experimental Design and Data Processing

Both parts (“human initiator” and “human responder”) in the experiment employed a 2 × 2 repeated measures design. In the human-initiator part, our first independent variable was the disposition of the robot (joint; disjoint), based on typical gaze-following behavior of the robot. Specifically, one identity (Jimmy – joint attention disposition) followed the participant’s gaze to the selected object in 80% of the trials, while the other identity (Dylan – disjoint attention disposition) followed the gaze in 20% of the trials. The second independent variable was online behavior, whether robot either followed or unfollowed the participant’s gaze in a trial. The dependent variable of interest in the human-initiator part of the task was latency of the return saccade to the robot’s face, calculated as this saccade’s onset time after the first fixation on the selected object. The independent variables in human responder part were disposition (joint; disjoint) and gaze-cue validity (valid; invalid). RT for target discrimination served as the dependent variable.

The RTs in both parts of the experiment were trimmed per cell (human initiator: joint-followed, joint-unfollowed; disjoint-followed, and disjoint-unfollowed; human responder: joint-valid, joint-invalid, disjoint-valid, and disjoint-invalid) per participant (+/-2.5 SD from the mean per cell). For the human-responder part, incorrect trials were discarded prior to RT-trimming. Statistical analyses were carried out in JASP (version 0.8.1.2; JASP Team, 2017).

Additionally, we examined whether joint attention disposition affected subjective ratings of each robot on the likeability and humanlikeness scales of the Godspeed questionnaire and we explored the role of autistic traits using the AQ. On the five questions in the experiment-related questionnaire, a qualitative analysis was carried out. Participants were divided in three categories (Jimmy, Dylan, no preference), based on the answer to question 1 (“Do you have a preference for one of the robots?”). Once these categories were made, Question 3 was analyzed looking at the adjectives that a participant used to describe the robots: three categories were created to classify them (human-like, neutral, and mechanical). People who used the same words to describe Jimmy and Dylan were considered in more detail, checking if they had a previous explicit preference (Question 1) and looking at the adjectives’ category. Question 2 (“Did you notice some differences? If yes, what kind of differences?”) and Question 4 (With whom would you prefer to interact with again? Why?”) were used to point out subjective perception about the robots’ behavior. These questions were analyzed as follows: considering the category of preference (Question 1), answers were divided in two groups (people who reported a perceived difference and people who did not). After that, the comments about the kind of differences were interpreted considering the most frequent comment’s topic (e.g., speed, comments referring to behavior, robot’s gaze direction). Finally, Question 5 was used to monitor participants’ expectations about the experiment.

## Results

### Human Initiator

**FIGURE 2:**
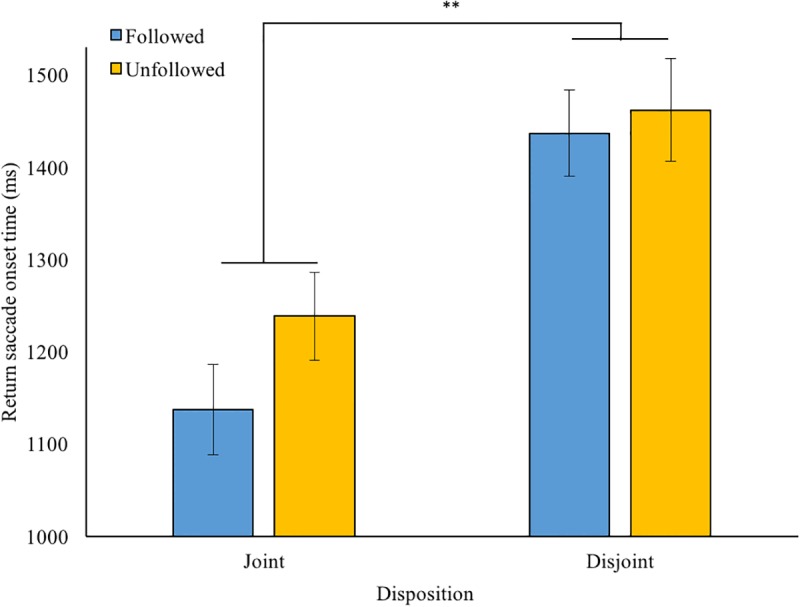
Return-to-face saccade latency (ms) after robot’s gaze response (followed or unfollowed) for each disposition condition (80% joint attention or 80% disjoint attention) in the gaze leading part. Error bars: +/-1 SE. ^∗∗^*p* < 0.01.

The data trimming method described above resulted in 2.9% of the data being discarded. The average saccade onset time following the sound cue was M = 324 ms (SD = 118). As we expected, there was no statistically significant difference between the joint and disjoint blocks (*p* = 0.36). On average, participants spent M = 1,629 ms (SD = 778) looking at the chosen object prior to returning their gaze to the robot’s face. Overall, this return saccade data did not follow assumptions of normality but was positively skewed (across the four conditions: mean *Z*_skewness_ = 2.92). Therefore, subsequent analyses were run on log_(base10)_-transformed data (mean *Z*_skewness_ = 0.53; normality assumed, no outliers). Below, we report untransformed descriptive statistics (see also **Figure 2**). The main analysis of the untransformed data, which pointed in the same direction, can be found in the Supplementary Material.

First, a 2 (disposition) × 2 (behavior) repeated-measures ANOVA revealed a main effect of disposition, *F*(1,31) = 11.3, *p* = 0.002, *η*^2^ = 0.27. That is to say, participants re-oriented their gaze quicker to the typically following robot [joint condition; M = 1,481 ms, SD = 661, 95% CI (1,252 1,710)] than to the typically unfollowing robot [disjoint condition; M = 1,776 ms, SD = 964, 95% CI (1,442 2,110)], see **Figure 2**. The main effect for online behavior failed to reach significance [*F*(1,31) = 3.35, *p* = 0.077, *η*^2^ = 0.10]: in trials where the robot followed the participant’s gaze, the mean return saccade onset was M = 1,603 [SD = 772, 95% CI (1,335 1,870)], while it was M = 1,654 ms [SD = 795, 95% CI (1,379 1,930)] when the robot did not follow. There was no interaction effect between disposition and online gaze-behavior; *F*(1,31) = 0.39, *p* = 0.54, *η*^2^ = 0.01. However, in exploration, paired samples *t*-tests suggested that the effect of gaze-following was prominent in the joint attention disposition condition (*p* = 0.023, one-tailed; Cohen’s *d* = 0.29) but not in the disjoint condition (*p* = 0.13; *d* = 0.12).

Additionally, we controlled for the mismatching numbers of trials in each level, concerning the 80/20% balance for following/unfollowing behavior within each disposition condition, as follows. From the two levels with the highest proportion of trials, we randomly selected a number of trials consistent with the number of valid trials in its counterpart level for each participant. For example, if for a certain participant 15 out of the 16 trials in the joint-unfollowed level remained after RT-trimming, a script randomly selected 15 joint-followed trials from the trimmed data. We repeated this simulation 10 times for each participant and conducted repeated-measures ANOVAs with the original disjoint-followed and joint-unfollowed values and each randomly selected mean for the other two levels. A total of 10 out of these 10 ANOVA’s confirmed the above significant main effect for disposition; with on average *F*(1,31) = 12.6, *p* = 0.001, *η*^2^ = 0.29, in which *p*-values < 0.001 were entered as *p* = 0.001. For following behavior, there was a significant main effect (α = 0.05) in 7 out of 10 simulations, average *F*(1,31) = 5.09, *p* = 0.055, *η*^2^ = 0.14. The interaction between these two factors was consistently not significant in any of the simulations (average *p* = 0.56).

There were no effects in relation to the AQ and return saccades in the human-initiator part (AQ as between-subjects variable grouped according to a median split all *p*s ≥ 0.12, *η*^2^s ≤ 0.01; as mean centered covariate all *p*s ≥ 0.22, *η*^2^s ≤ 0.05) – the latter covariate values with the exception of a non-significant main effect for AQ: *F*(1,30) = 3.77, *p* = 0.062, *η*^2^ = 0.11. In other words, AQ correlated positively but not significantly with return saccade RT (*r* = 0.33, *p* = 0.062), such that those with higher AQ score took longer to look back to the robot’s face overall.

### Human Responder

While testing one participant, technical difficulties arose during the human-responder part, which occurred after the midway break in both the joint and the disjoint attention disposition conditions. We presented the remaining block immediately after these difficulties occurred. To preserve counterbalanced outcomes in our analysis, we chose to include the data of this participant. Excluding this participant’s data from our analysis altogether had no impact on the results reported below.

**FIGURE 3:**
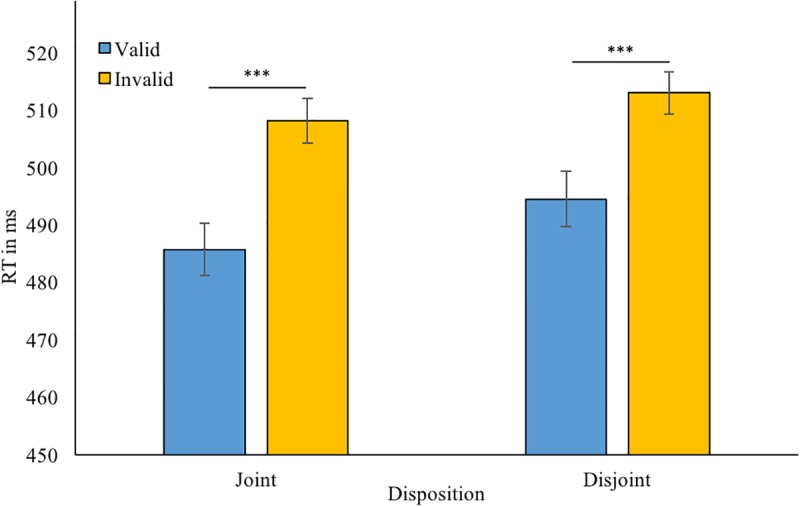
Mean reaction times in the gaze-cueing part for validly and invalidly cued trials for each robot: 80% followed in the previous gaze leading part (joint attention disposition); 80% unfollowed (disjoint attention disposition). Error bars = +/-1. ^∗∗∗^*p* < 0.001.

Rejection of incorrect trials and RT trimming resulted in discarding 5.0% of the data. A 2 (disposition) × 2 (cue validity) ANOVA was conducted on the remaining data, which was found to be normally distributed. We found a main effect of validity; *F*(1,31) = 40.4, *p* < 0.001, *η*^2^ = 0.57. Participants showed shorter RTs for validly cued targets [M = 490 ms, SD = 59, 95% CI (473 506)] than for invalidly cued targets [M = 511 ms, SD = 66, 95% CI (491 529)]. However, there was neither a main effect of disposition (joint/disjoint; *p* = 0.34, *η*^2^ = 0.03) nor an disposition × validity interaction; *p* = 0.49, *η*^2^ = 0.02 (**Figure 3**).

Remarkably, AQ score interacted with robot disposition in the human-responder part (mean-centered covariate *p* = 0.012, *η*^2^ = 0.19; median split between-subjects variable *p* = 0.008, *η*^2^ = 0.20). *Post hoc* paired samples *t*-tests showed that participants below the AQ median showed no RT difference between the joint (M = 500 ms, SD = 69) and disjoint attention condition (M = 511 ms, SD = 78); *p* = 0.33; *d* = 0.25. However, those in the high AQ group were significantly faster for the joint attention disposition (M = 483 ms, SD = 48) than for the disjoint attention disposition (M = 508 ms, SD = 62); *t*(15) = 3.92, *p* = 0.001, *d* = 0.98. This was confirmed by an additional analysis, in which we subtracted mean RTs in the joint condition from those in the disjoint condition and found that higher AQ scores correlated positively with this contrast (*r* = 0.44, *p* = 0.011; after removal of one outlier (*Z* = -3.76) in the difference scores *r* = 0.53, *p* = 0.002). No other main effects or interactions involving the AQ and human-responder were found (all *p*s ≥ 0.11; η^2^s ≤ 0.08).

### Subjective Ratings

One-tailed Wilcoxon tests indicated that participants rated the joint robot more human-like (Mdn = 15) than the disjoint robot (Mdn = 13; Godspeed, anthropomorphism subscale; *Z* = 3.06, *p* = 0.001) and more likeable (Godspeed, likeability subscale; *Z* = 1.68, *p* = 0.046, one tailed; joint Mdn = 18, disjoint Mdn = 17.5). There were no significant correlations between the AQ and Godspeed scores.

### Qualitative Analysis of Additional Questions

A total of 15 of our 32 participants indicated an explicit preference for Jimmy (joint attention disposition), 13 indicated no preference for either, and 4 explicitly preferred Dylan (disjoint attention disposition), from which we conclude that the robot with the disjoint behavior is perceived as less pleasant and less likely to be preferred^1^. Out of the 13 participants who had no-first preference here, 11 chose a robot for the second interaction (Question 4 “With whom would you prefer to interact with again? Why?”; Jimmy: 6, Dylan: 5) and only 2 confirmed a neutral preference; 18 people preferred to interact with Jimmy again, 12 with Dylan, and 2 with neither. The most commonly reported reason for the choice made in the second interaction for Jimmy is that he was perceived as more cooperative, more reliable and more pleasant. For Dylan, comments mostly refer to his “challenging behavior,” or his “competitiveness.”

Further, only 6 participants did not notice any differences between the two robots (question “Did you notice some differences? If yes, what kind of differences?”). The other 26 participants did notice differences and we included them in the following categories: gaze’s speed (4 participants), gaze movement (13 participants), behavioral comments (7 participants), and mentalizing comments (2 participants). Gaze movement’s comments were mostly expressed as follows: “Jimmy is looking at the object that I am looking at, Dylan is not following me”; behavioral comments were mostly expressed as follow: “Jimmy is doing what I do, Dylan is behaving on its own”; gaze speed comments mostly referred to the perception that one of the robots was faster or slower. Finally, two people used mentalizing words to describe the perceived difference: one said that Jimmy was more misleading than Dylan, the other said that Dylan was more demanding.

Question 3 required participants to describe both Jimmy and Dylan with one adjective each. We classified these into mechanical (e.g., “mechanic,” “artificial”), neutral (e.g., “neutral,” “normal”), and human-like adjectives. We further classified the human-like adjectives into positive and negative responses (e.g., “cute,” “reliable,” and “loyal” as positive and “unreliable,” “not cute,” and “unpleasant” as negative). Response-frequencies of this classification are found in **Table 1**.

**Table 1:** Response-classification frequencies to “describe each robot in one adjective.”

Classification		Jimmy	Dylan	Same adjective category for both	Same adjective for both
Human-like	Total	21	19	14	4
	positive	15	7		
	negative	6	12		
Neutral		5	5	3	3
Mechanical		6	8	5	4

For the human-like responses, we compared the positive and negative adjective categories for each robot, χ^2^ = 4.8, *p* = 0.028. This indicates that indeed participants attributed proportionally more positive traits to the robot with the joint disposition and negative traits to robot with the disjoint disposition.

## Discussion

This study examined the implicit and self-reported effects of successful versus unsuccessful initiation of joint attention with a robot avatar.

### Human-Initiator Effects

First, during the human-initiator part of our study, we found that return saccades to robot faces who typically follow our gaze are faster than toward robot faces who typically look elsewhere. This replicates the findings from Bayliss et al. (2013) and indicates that it is easier for us to re-orient our attention to those with whom we establish joint attention relative to those with whom we do not, including humanoid robots. Our study extends previous findings by showing that attribution of identity (“those who follow my gaze” versus “those who do not follow”) can occur merely through observed/learned behavior, and not necessarily through physical appearance, as in our study we presented the exact same robot face, with just different probabilities of following gaze of participants. Furthermore, and important to note, we showed that effect was due to attributed disposition of the robot, and not necessarily to its trial-by-trial online behavior, although the latter statement needs to be treated with caution, as the main effect of contingent behavior was significant in 7 out of 10 analyses with equal numbers of trials. In either case, it appears that we accumulate and retrieve identity information, and engage attention toward an agent, based on the attributed trait (learned – perhaps even implicitly – through experience of interaction with the agent). Seeing that these findings were not reversed between the two dispositional identities, we can confidently conclude that the difference in return saccade onset times do not merely reflect responses to odd-balls, or violations of expectations *per se*. Furthermore, this indicates that these mechanisms do not reflect easier disengagement from the simultaneously attended object based on both participant and robot keeping watch of it.

While we explain this faster reorienting the gaze to the avatar to facilitated engagement with those with whom we establish joint attention, it is possible that lower-level mechanisms such as imitation rather than joint attention underpin these faster return saccades. Studies have shown that gaze is biased toward those who mimic our expressions (Neufeld and Chakrabarti, 2016) and that this mimicry leads to reward-related neural response (Hsu et al., 2017). More related to actions rather than facial expressions, it has been found that the anticipation of being imitated facilitates subsequent motor performance (Pfister et al., 2013). Our findings could therefore potentially be explained by a facilitation of the required task actions by mere imitation of the participant. Nonetheless, as joint attention is at the least an implicit result of this imitation, these explanations are not mutually exclusive.

### Human-Responder Effects

We found no significant differences in the gaze-cueing effect between the two dispositions. In response to the robot that typically followed participants’ gaze, a gaze-cueing effect was of similar magnitude than in response to the robot in the disjoint condition. This does not replicate the findings from Dalmaso et al. (2016). We identify two noticeable differences between their study and ours. First, in their experiments, participant’s gaze location in the joint attention initiator task was determined by task instructions, whereas in ours it was the participant’s choice. In more general terms, task instructions, by their nature, limit the aspects of behavior and performance which can be studied (Gozli, 2017). Perhaps attentional orienting in subsequent interaction is different when the avatar violates task instructions during previous interaction rather than disagreed with the participant’s choice. Second, different human faces were used by the authors, which potentially allowed for deeper encoding of person information than using physically same robot stimulus with only a different name and behavior. Both Dalmaso et al.’s. 2016 experiments and the presented work use non-predictive gaze-cueing. However, it is possible that the lack of identity encoding at the face-level further contributed to our lack of findings. Moreover, participants experienced that our avatars could easily be “reprogramed.” In addition, perhaps purely gaze behavior is too subtle to greatly affect interaction, which might have been encouraged by other factors such as overall appearance of the robot. In fact, participants described the robot as cute, also the one with the disjoint attention disposition.

Nevertheless, although the attributed identity of the robot did not affect gaze-cueing, it was sufficient to affect subjective ratings of anthropomorphism and likeability, as well as indications of which robot the participants preferred as suggested by our interpretation of the additional questions. In reflection, the Godspeed items (Bartneck et al., 2009) we used appear to reflect physical characteristics more than behavioral attributions, which complicates a clear replication of previous findings that we have a preference for those who follow our attention (Bayliss et al., 2013; Grynszpan et al., 2017). Additionally, perhaps the fact that gaze-cueing was non-predictive for both identities in the human-responder part attenuated clear preferences for either, as potentially illustrated by the 15 no-preference responses. Regardless, we carefully propose that agents with whom we successfully establish joint attention are preferred to those who do not follow our gaze, and are perceived as more human-like and likeable.

Our participants were all in a low AQ-range. The findings suggest social aptitude-related difficulties in social orienting. Notably, those with relatively high AQ scores were slower in both joint attention tasks. As the initiator, they showed a delayed re-orienting to the robot than those with fewer autistic-like traits, independent of whether they were interaction with the joint or with the disjoint robot. This displays a partial match with a previous study, in which a correlation between autistic-like traits and attentional orienting toward faces was only cautionally reported for the faces that followed the participant’s gaze (Edwards et al., 2015). As responders, our participants were slower with the disjoint robot than with the joint robot. Unlike Dalmaso et al. (2016), where participants were less cued by joint faces, our effect occurred regardless of cue validity. As past findings for autistic traits in gaze perception have been mixed (Frischen et al., 2007), we can only interpret the above findings with caution.

Regarding the qualitative data it appears that, even if participants interacted only with images of the iCub, they were quite involved. This phenomenon could be attributed to two factors:

(1) iCub’s physical characteristics: the robot’s size and appearance is like a 5-year-old child; this could bring people to interact with it in a child-related frame of mind.
(2) iCub’s names and personalities: presenting the robots as having different names and different personalities, could have enhanced anthropomorphism, allowing participants to use more human-like words.

Further, it appears that the robot in the joint condition is more likely to be perceived as more cooperative and pleasant, leading people to use more positive adjectives. The robot with the disjoint attention disposition seemed to be perceived as more challenging and less likely to be chosen for a second interaction, in favor of the robot with the disposition to respond with joint attention.

In terms of future directions, the fact that differences in return saccade onset times were larger per blocked condition (joint-disjoint attention disposition) than between trials (followed-unfollowed online behavior) is reassuring to design more naturalistic experiments. Using a physical, humanoid robot such as iCub (Metta et al., 2010) can create more realistic social interaction scenarios, while maintaining experimental control (cf. Wykowska et al., 2016). On the other hand, it does not allow to present different identities randomly between trials. Nevertheless, the current study confirms that it is possible to utilize a blocked design, in which participants can be led to believe that the robot is “reprogramed” in between the different blocks. These results can have a significant impact on design of robots who are to enter human social sphere in the near future. In particular, it is seems recommendable to equip robots with certain “traits,” and one of them is likelihood of following/responding to human’s bids for attention. Such robots would evoke larger degree of engagement, and would be perceived more human-like and likeable – important aspects of future social robots.

Taken together, we have demonstrated that human–robot gaze interaction is in line with phenomena observed in human–human interaction. Orienting attention to robots who accept our invitation to joint attention is facilitated over to those who do not. Even though it remains unclear whether this affects our responding to the robot’s bidding for attention, it appears that we perceive those with whom we successfully initiate joint attention as more human-like and perhaps more likeable: we prefer robots who reliably look where we prefer to look.

## Author Contributions

CW designed and programmed the experiment, carried out the quantitative analyses, and wrote the manuscript with the exception of the sections specified herein. SM wrote the Section “Materials and Methods,” analysed the qualitative data, and reported and discussed the results of the qualitative analysis. AW made substantial contributions to the design of the study, provided considerable intellectual content, contributed to writing up the manuscript, and revised the work critically. All authors provided approval of the version to be published and agreed to be accountable for all aspects of the work in ensuring that questions related to the accuracy or integrity of any part of the work are appropriately investigated and resolved.

## Conflict of Interest Statement

The authors declare that the research was conducted in the absence of any commercial or financial relationships that could be construed as a potential conflict of interest.

## Footnotes

1 When we explored the gaze-cueing effects in the human-responder task as a function of the group which participants belonged to (explicit preference for Jimmy or no explicit preference for Jimmy), no significant interaction was found, see Supplementary Material.

## Supplementary Material

The data related to this study can be accessed online at https://github.com/s4hri/engagement-with-robots-that-follow-gaze. doi: 10.5281/zenodo.1160396
