# Supplementary material for: Robot Faces that Follow Gaze Facilitate Attentional Engagement and Increase Their Likeability
Source: Front Psychol. 2018 Feb 5;9:70. doi: 10.3389/fpsyg.2018.00070 (PMC5807394; doi:10.3389/fpsyg.2018.00070)
Supplement: Supplementary file 1 [file Presentation_1.PDF]

Supplementary Material

Robot faces that follow gaze facilitate attentional engagement and increase their likeability

Cesco Willemse[1], Serena Marchesi[1], Agnieszka Wykowska[1*]

[1]Social Cognition in Human-Robot Interaction, Istituto Italiano di Tecnologia, Genoa, Italy

*** Correspondence:**
Agnieszka Wykowska
agnieszka.wykowska@iit.it

Supplementary analysis
In the main text, we transformed the return-saccade data from the human-initiator part to meet the assumptions of normality. Here, we report the results of the main analysis (previously, untransformed descriptives were already reported to interpret the direction of the effects). This 2 (identity) X 2 (behaviour) repeated-measures ANOVA revealed a main effect of identity, $F(1, 31) = 9.08$, $p = .005$, $\eta^2 = .23$. Participants re-oriented their gaze to the robot's face quicker in the joint condition than in the disjoint condition. However, a main effect for identity was not significant; $F(1, 31) = 2.60$, $p = .12$, $\eta^2 = .08$. Again, there was no interaction effect between identity and online gaze-behaviour; $F(1, 31) = 0.89$, $p = .35$, $\eta^2 = .03$. To match our main analysis described in the main text, we carried out paired samples t-tests for each disposition to compare followed and unfollowed return-saccade RTs: joint $p = .063$, disjoint $p = .79$.

Additionally, we reported that 15 of the 32 participants had an explicit preference for Jimmy (joint attention disposition), 13 had no preference and 4 preferred Dylan (disjoint attention disposition). For exploratory purposes, we conducted an analysis for the human respomnder task on the basia of explicit preference as a between-subject factor with two levels (explicit preference for Jimmy; no explicit preference for Jimmy). However, this yielded no statistically significant results in addition to the subsisting main effect of validity (all ps $\geq .15$, η^2s $\leq .07$).

Supplementary tables

Supplementary Table 1.

Additional questions asked to participants at the end of the experimental session.

	Question
1-	Do you have a preference for one of the robot?
2-	Did you notice any difference between them? If yes, what kind of difference?
3-	How would you describe them with one adjective for each?

4- With whom would you prefer to interact with again?

5- What do you think is the purpose of this experiment?

Supplementary figures

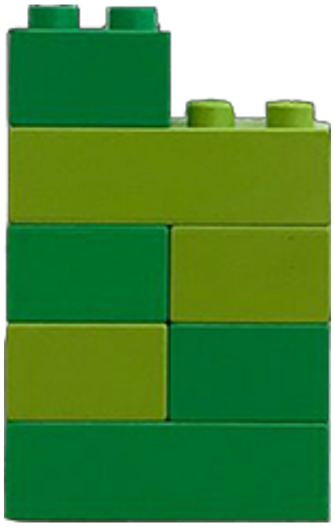

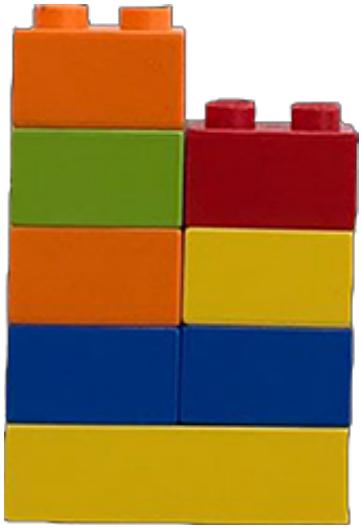

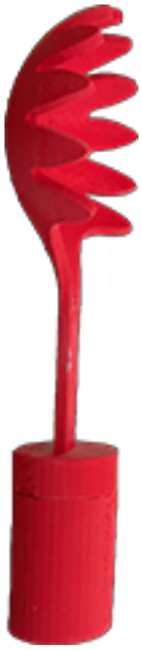

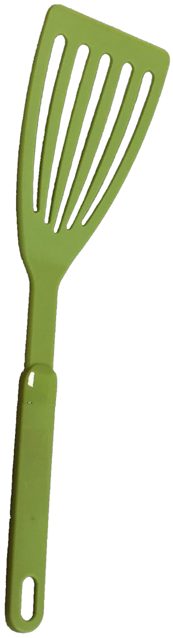

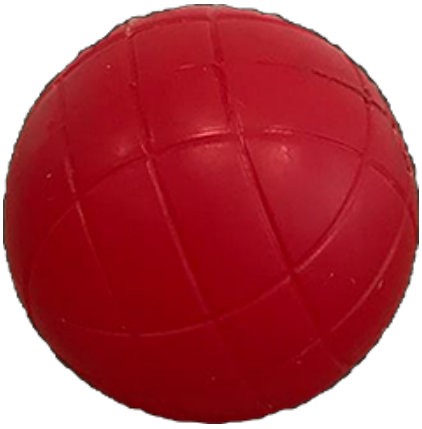

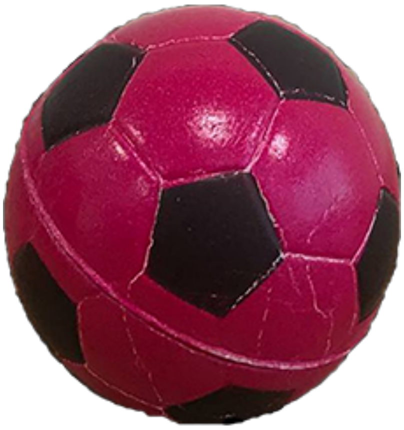

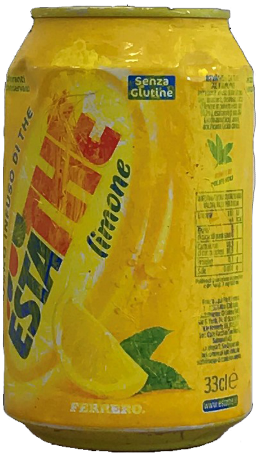
Senza
Glutine
ESTATHE
limone
33cl
FERRERO

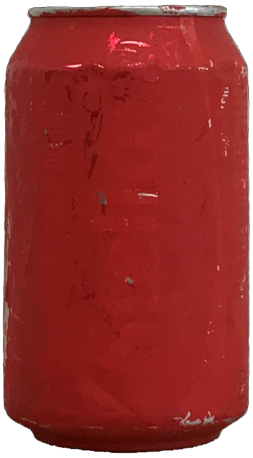

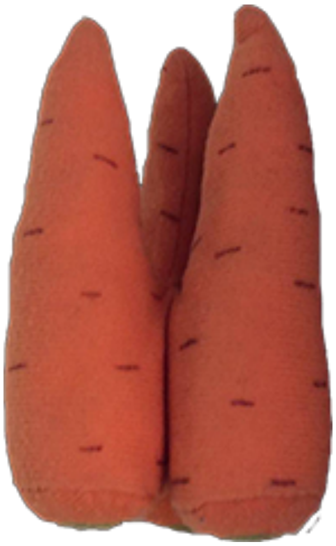

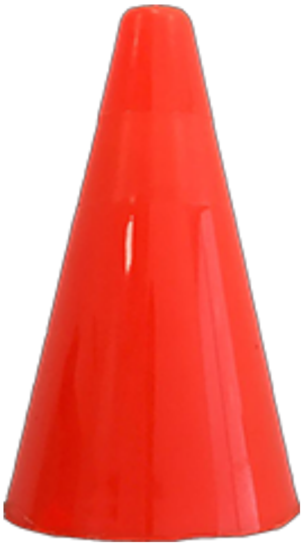

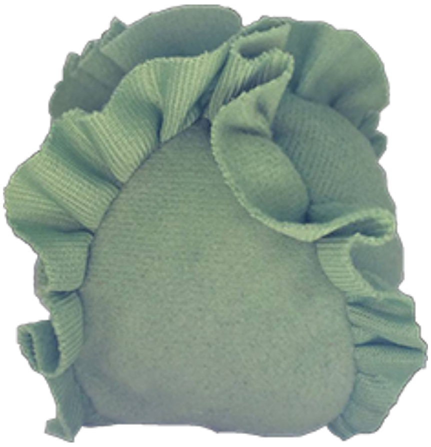
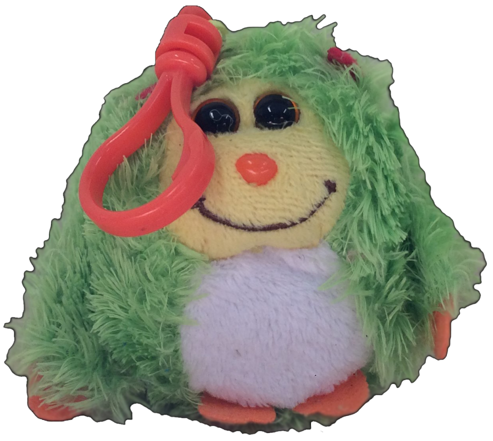
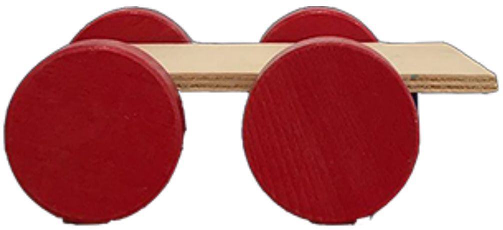
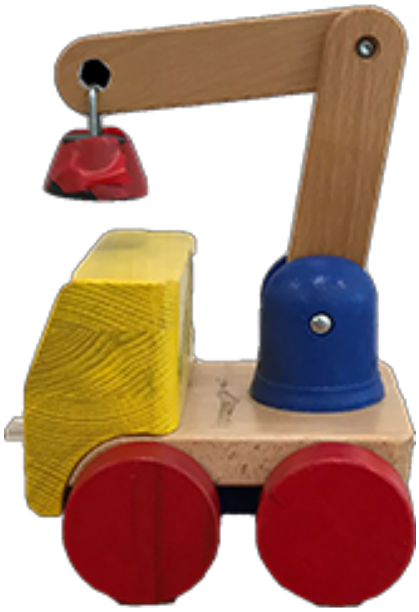
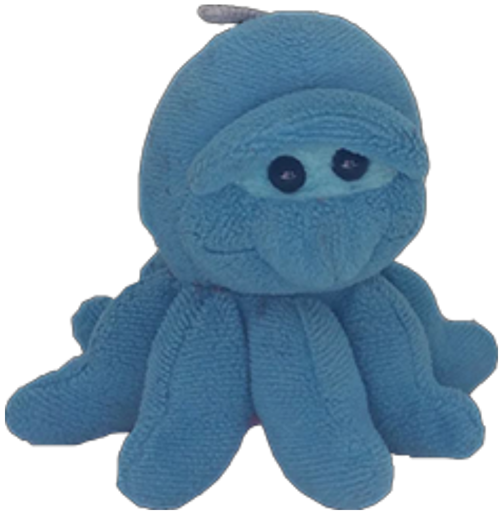
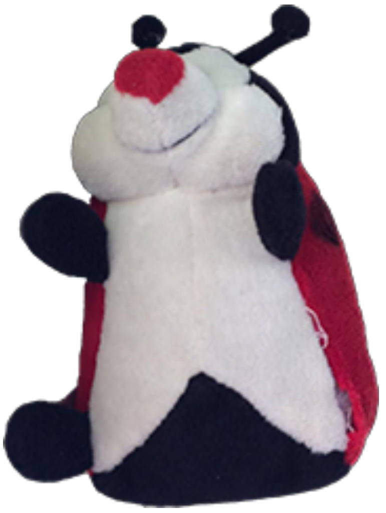

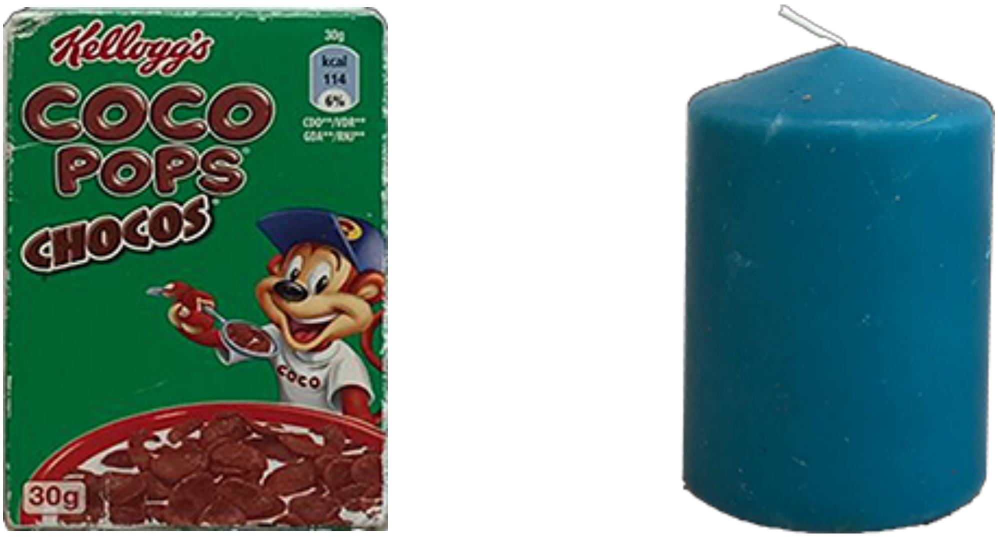

Supplementary Figure 1. Object pairs used in the human-initiator task. Image location (left or right) was counterbalanced between trials. The last pair was only used in the practice session. Note: not drawn to scale.
